# Complete Genome Sequence of *Geobacillus thermoglucosidasius* NCIMB 11955, the Progenitor of a Bioethanol Production Strain

**DOI:** 10.1128/genomeA.01065-16

**Published:** 2016-09-29

**Authors:** Lili Sheng, Ying Zhang, Nigel P. Minton

**Affiliations:** Clostridia Research Group, BBSRC/EPSRC Synthetic Biology Research Centre (SBRC), School of Life Sciences, Centre for Biomolecular Sciences, University of Nottingham, University Park, Nottingham, United Kingdom

## Abstract

The industrially important thermophile *Geobacillus thermoglucosidasius* has the potential to produce chemicals and fuels from biomass-derived sugar feedstocks. Here, we present the genome sequence of strain NCIMB 11955, the progenitor of an ethanologenic industrial strain, revealing 11 single-nucleotide polymorphisms and 2 indels compared to strain DSM 2542 and two novel plasmids.

## GENOME ANNOUNCEMENT

*Geobacillus thermoglucosidasius* is a Gram-positive, thermophilic, spore-forming bacterium that performs a typical mixed-acid fermentation (1). It has biotechnological potential for chemical and fuel generation from biomass-derived feedstocks (2–4), as illustrated by the creation of TM242, which produces bioethanol at yields close to theoretical maxima (5). The genome sequence of strain DSM2542, originally isolated in Japan (6, 7), has already been published (8). However, as phenotypes vary widely within the same strain due to genotypic changes (9, 10), we have sequenced the direct progenitor of TM242, NCIMB 11955.

Genomic DNA was prepared using phenol-chloroform extraction. Paired-end libraries were sequenced using an Illumina MiSeq bench-top sequencer (Deepseq, University of Nottingham, Nottingham, UK), producing 1.66 Gb of data consisting of a total of 801,181,830 nucleotides (3,203,788 reads with an average length of 250.1 bp). Using CLC Genomics Workbench version 7.0.4 (CLCbio), 3,203,678 reads were produced through trimming with a quality score limit of 0.05 and maximum ambiguous nucleotide of 2. The genome sequence was then derived by mapping to DSM2542 (CP012712) (using 80% as the cutoff for single-nucleotide variant calling) as a reference and *de novo* assembly.

The entire reference was covered by 92% of the reads (average coverage of 189.38 ± 41.51) with four gaps (699, 253, 66, 321 bp). While the first three were closed through identification of *de novo* assembled contigs, the 321-bp gap was closed through PCR amplification and Sanger sequencing of the missing region. A total of 11 single-nucleotide polymorphisms (8 nonsynonymous) and 2 indels were identified compared to DSM 2542. Affected genes included those encoding an MFS transporter (AOT13_RS03255, V429A), rpoE (AOT13_RS03625, A106V), a transposase (AOT13_RS05685, T145M), YggS (AOT13_RS08685, stop: 83R), CodY (AOT13_RS09075, A143V), CheA (AOT13_RS09210, V322D), RimP (AOT13_RS09295, K137 fs), and adenine phosphoribosyltransferase (AOT13_RS17360, P65T).

BLAST analysis of two unmapped contigs designated pNCI001 (83,925 bp, 43.5% GC) and pNCI002 (47,893 bp, 39.0% GC), revealed homology to a number of *Geobacillus* plasmids. Plasmid pNCI001 shared homology with pGS18 (AM886060: 55% coverage, 98% identity) and pLW1071 (CP000558: 64% coverage, 98% identity), whereas plasmid pNCI002 was homologous to pGG01 (DQ146476: 23% coverage, 98% identity), pGEOTH01 (CP002836: 66% coverage, 99% identity), and pGY4MC101 (CP002294: 65% coverage, 99% identity).

Genome annotation was carried out by the NCBI Prokaryotic Genome Annotation Pipeline (PGAP) service, resulting in a total of 3,844 coding sequences (3,708 on the chromosome, 81 on pNCI001, and 55 on pNCI002). The chromosome shared identical features to that of DSM 2542 (8). The plasmids carry mostly hypothetical proteins. Open reading frames responsible for replication of the plasmids were identified as RepB (BCV53_19210, BCV53_19380) for pNCI001 and RepA (BCV53_19650), both of which belong to the Rep3 superfamily, suggesting a rolling-circle mode of replication (11, 12). The mapping coverage (294× and 585×, respectively) suggests that the copy number of pNCI002 is twice that of pNCI001. The latter encodes a number of proteins of predicted metabolic relevance, including acyl-CoA dehydrogenase (BCV53_19405), flavin reductase (BCV53_ 19410), and acetaldehye dehydrogenase (BCV53_19435). However, no proline- and hydroxyproline-related metabolic genes were predicted, which is in contrast to pGEOTH01 present in *G. thermoglucosidasius* C56-YS93 (13).

### Accession number(s).

The complete genome sequence has been deposited in GenBank under the accession numbers CP016622 (chromosome), CP016623 (pNCI001), and CP016624 (pNCI002).
